# An aggressive systemic mastocytosis preceded by ovarian dysgerminoma

**DOI:** 10.1186/s12885-020-07653-z

**Published:** 2020-11-27

**Authors:** Makiko Tsutsumi, Hiroki Miura, Hidehito Inagaki, Yasuko Shinkai, Asuka Kato, Takema Kato, Susumu Hamada-Tsutsumi, Makito Tanaka, Kazuko Kudo, Tetsushi Yoshikawa, Hiroki Kurahashi

**Affiliations:** 1 Division of Molecular Genetics, Institute for Comprehensive Medical Science, Fujita Health University, 1-98 Dengakugakubo, Kutsukake-cho, Toyoake, Aichi 470-1192 Japan; 2 Department of Pediatrics, Fujita Health University School of Medicine, Toyoake, Japan; 3 ITOCHU Collaborative Research-Molecular Targeted Cancer Treatment for Next Generation, Graduate School of Medicine, Nagoya University, Nagoya, Japan; 4 Department of Virology and Liver Unit, Nagoya City University Graduate School of Medical Sciences, Nagoya, Japan

**Keywords:** Aggressive systemic mastocytosis, *KIT*, Dysgerminoma, Germ cell tumor, *TP53*, Loss of heterozygosity

## Abstract

**Background:**

Aggressive systemic mastocytosis (ASM) is a rare malignant disease characterized by disordered mast cell accumulation in various organs. We here describe a female ASM patient with a previous history of ovarian dysgerminoma.

**Methods:**

Molecular cytogenomic analyses were performed to elucidate an etiological link between the ASM and dysgerminoma of the patient.

**Results:**

This patient was affected by ovarian dysgerminoma which was treated by chemotherapy and surgical resection. Having subsequently been in complete remission for 2 years, she developed symptoms of ASM. A somatic D816A mutation in the *KIT* gene was detected in her bone marrow, which facilitated the diagnosis of ASM. Unexpectedly, this *KIT* D816A variant was also detected in the prior ovarian dysgerminoma sample. Whole-exome sequencing allowed us to identify a somatic nonsense mutation of the *TP53* gene in the bone marrow, but not in the dysgerminoma. Microarray analysis of the patient’s bone marrow revealed a copy-number-neutral loss of heterozygosity at the *TP53* locus, suggestive of the homozygous nonsense mutation in the *TP53* gene. In addition, the loss of heterozygosity at the *TP53* locus was also detected in the dysgerminoma.

**Conclusions:**

These results indicated that either the mast cells causing the ASM in this case had originated from the preceding ovarian dysgerminoma as a clonal evolution of a residual tumor cell, which acquired the *TP53* mutation, or that both tumors developed from a common cancer stem cell carrying the *KIT* D816A variation.

## Background

ASM is one of the advanced forms of systemic mastocytosis (SM) with a poor prognosis. In this disorder, clonal mast cells become abnormally accumulated in the skin, lymph nodes, liver, gastrointestinal tract and bone marrow (BM) where they are activated and release mediators such as histamine, tryptase and cytokines that then cause organ damage [1–3]. The prevalence of ASM is 0.09 per 100,000, and the median age at diagnosis is over 60 years. ASM is quite rare in pediatric population [4–6]. The D816V mutation in *KIT* is frequently found in the tumor cells of SM patients and is an important part of the established diagnostic criteria for ASM. In addition to *KIT* variations, somatic mutations in other genes also occur in ASM that facilitate tumor growth [2, 7].

Ovarian dysgerminoma is one of the common malignant germ cell tumors believed to develop from primordial germ cells (PGCs) due to its morphology. These malignant tumors more frequently occur in adolescents and young adults, and surgical resection accompanied with chemotherapy generally result in a good prognosis [8, 9].

In our present case report, we describe an adolescent case of ASM in a female with a previous history of ovarian dysgerminoma. Genetic analysis indicated a common origin for these malignancies and provided insights into the processes underlying the progression to ASM.

## Methods

### Samples for genetic analyses

Genomic DNA was isolated from peripheral blood (PB) and BM samples of the study patient using the QuickGene DNA whole blood DNA kit L (Kurabo, Osaka, Japan). Genomic DNA of the buccal mucosa was extracted using the DNeasy Blood and Tissue kit (Qiagen, Tokyo, Japan). A formalin-fixed paraffin-embedded specimen of the surgically dissected ovarian dysgerminoma was deparaffinized in xylene followed by proteinase treatment, phenol/chloroform extraction and ethanol precipitation of DNA. Conventional G-banding of the patient’s bone marrow was performed using a standard method.

### PCR amplification and sequencing

DNA fragments were amplified by PCR using Ex-Taq or LA-Taq polymerase (Takara, Kusatsu, Japan) followed by direct sequencing with the primers listed in Table S1 of the Additional file 1. Where indicated, PCR products were cloned into the pT7 Blue T-vector (Novagen, Madison, WI, USA) and then sequenced.

### Real-time quantitative PCR

Quantitative PCR of the *KIT* gene was performed on the StepOnePlus Real-Time PCR system (Thermo Fisher Scientific, Waltham, MA, USA) using the PowerUp SYBR Green Master Mix (Thermo Fisher Scientific) with the primers listed in Table S1 of the Additional file 1. The *DROSHA* gene was used as an internal control.

### Whole-exome sequencing

Whole-exome sequencing of the PB and BM specimens was performed as described previously [10]. The sequencing data were analyzed with Variant Studio 2.3 (Illumina, San Diego, CA, USA), Integrative Genomics Viewer ver.2.4.19 (Broad Institute, Cambridge, MA, USA) and Mutect2 software (Broad Institute). The list of known cancer genes in the Cancer Gene Census [11] was used to identify mutations in the study patient.

### Cytogenetic microarray

Whole genomic microarray analysis of the BM sample was performed using the CytoScan 750 K array (Affymetrix, Santa Clara, CA) and analyzed using R package Rawcopy [12].

## Results

### Case presentation

The study case was affected with ovarian dysgerminoma when she was 13 years old. She received 4 cycles of chemotherapy, consisting of bleomycin, etoposide and cisplatin, followed by complete surgical resection of the tumor. After this resection, the patient’s α-fetoprotein (AFP) level fell from 1053 ng/ml (normal range, 0–10.5 ng/ml) to normal levels and she received 2 cycles of postoperative chemotherapy consisting of carboplatin and etoposide.

**Fig. 1:**
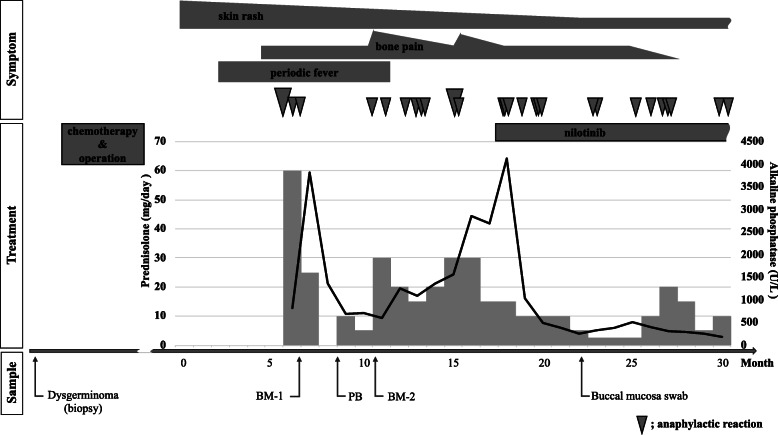
Clinical course of the study patient, and timeline for the tissue and blood sampling for genetic analysis. The black line and gray bar graphs indicate the kinetics for the alkaline phosphatase disease biomarker and daily prednisolone requirement, respectively. The gray inverse triangles indicate the occurrence of an anaphylactic reaction, with the severity denoted by the size of the symbol

She had been in complete remission for 2 years but developed recurring episodes of skin rash, bone pain, periodic fever and anaphylactic reactions when she was 16 years old (Fig. 1). A computed tomography scan suggested skeletal involvement with osteosclerosis mainly affecting the spine and osteolysis in a limited area of bones, but no tumor mass was observed suggesting that ovarian dysgerminoma recurrence was unlikely. BM examination of the osteolytic lesions revealed multifocal, dense infiltrates of mast cells that showed positive immunohistochemical staining for mast cell tryptase, CD25, CD33, and c-KIT, but no dysgerminoma cell was observed. A mutation at codon 816 of the KIT gene was further revealed in these cells, as detailed later. In addition, the serum tryptase level was markedly elevated at 276 μg/L (normal range, 1–15 μg/L), but AFP level was normal. The patient was subsequently diagnosed with ASM in accordance with the 2016 WHO classification of mastocytosis. The initial therapeutic intervention, including prednisolone, and histamine H1- and H2- receptor antagonists, improved her general condition, but the frequency of anaphylaxis did not decrease significantly and she became steroid dependence. Furthermore, her serum alkaline phosphatase level, which is indicative of disease activity, temporarily decreased and then rose again from 595 to 2857 U/L (normal range, 115–359 U/L). Mast cell accumulation in the BM was reevaluated before applying a second-line therapy. At that time, the proportion of the mast cells identified by c-KIT staining had decreased to 5% of all nucleated cells. The karyotype of the BM was normal (46,XX). After introducing nilotinib, a potent tyrosine kinase inhibitor for patients harboring a *KIT* codon 816 mutation, her clinical symptoms that had been occurring on an almost daily basis improved moderately and the prednisolone dose could therefore be reduced. Her serum alkaline phosphatase level was also normalized. However, she still suffered with anaphylactic reactions almost once per month, which suggested the existence of uncontrolled residual and reactive mast cells.

### Detection of a KIT mutation

**Fig. 2:**
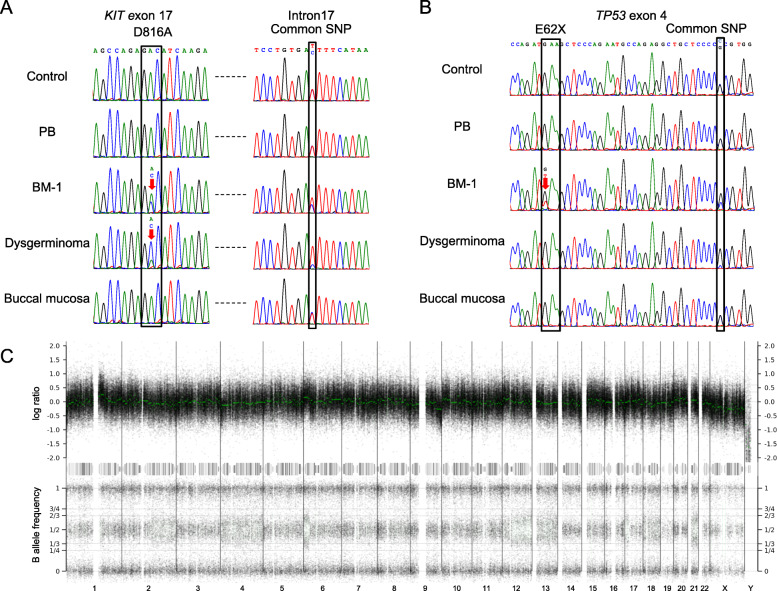
Genetic analyses of the study patient. **a**, **b** PCR-direct sequencing of the mutation sites for *KIT* (**a**) and *TP53* (**b**). PB from a healthy volunteer was used as a control. **c** Microarray-analyzed whole genome view of the BM-1 sample. The top row indicates the log ratio. The median log ratio of each segment is shown in green. The bottom row shows the B allele frequency. The chromosomes are indicated below the panel

To make a definitive diagnosis of ASM in our study patient, the codon 816 region of the *KIT* gene was amplified by PCR and directly sequenced (Fig. 2a; Additional file 2: Fig. S1A). A somatic mutation, D816A (NM_000222.2:c.2447A > C, COSM24675) was identified in the patient’s BM that had been sampled at the onset of the ASM-associated symptoms (BM-1). This mutation was not detectable in the PB, nor in the BM obtained after the initial treatment (BM-2). The percentage of mast cells in the BM-1 and BM-2 smears determined by microscopic examination was 50 and 5%, respectively. The variant allele frequency (VAF) in the BM-1 sample was 0.34, which may have been due to contamination by normal cells.

D816 mutations in the *KIT* gene are common to germ cell tumors [13] and we thus tested a biopsy specimen that was obtained from the dysgerminoma lesion in our study case prior to chemotherapy. The same D816A *KIT* mutation was detected. Notably also, the mutant allele ratio in the dysgerminoma was considerably higher than that of the normal allele (VAF = 0.75). *KIT* gene amplification was not evident by quantitative PCR (Additional file 2: Fig. S1B), suggesting that the normal allele had been deleted in the dysgerminoma cells. Indeed, the ratio between the two alleles of a heterozygous common single nucleotide polymorphism (SNP), C > T (rs1008658), located 115 bp downstream of the mutation site, was also skewed in samples carrying the D816A variant. PCR products including both the *KIT* D816 and rs1008658 sites were cloned and sequenced individually (Additional file 2: Fig. S1C). The *KIT* D816A mutation was found to be linked to the T allele of the SNP. Since the VAF in the BM-1 sample was 0.64, the normal allele was thought be also lost in the ASM cells.

### Genome-wide analysis of genetic alterations

We next explored the additional mutations associated with ASM development in our patient as ASM cells often carry mutations in cancer-related genes in addition to *KIT* [2]. Whole-exome sequencing of the genomic DNA from BM-1 and PB samples was performed and revealed a nonsense mutation in *TP53* and a missense mutation in *TET2* (Additional file 1: Tables S2 and S3). The nonsense mutation in *TP53*, E62X, (NM_000546:c.184G > T) was detected in BM-1, but not in the dysgerminoma nor the other samples (Fig. 2b). Normal cell contamination prevented us from determining the status of the normal allele (VAF = 0.31). When we examined for the presence of a common heterozygous SNP (rs1042522), located 31 bp downstream of the mutation site, an allelic imbalance was found in the BM-1 cells (Fig. 2b). Next-generation sequencing reads demonstrated that the mutant allele was present in the G allele of the SNP (Additional file 3: Fig. S2). From this phasing data, loss of heterozygosity (LOH) at this locus was evident in the BM-1 sample, suggesting a biallelic inactivation of the *TP53* gene in the ASM cells. To next determine whether the normal allele of the *TP53* gene had been deleted, its copy number was analyzed by whole-genomic microarray. LOH spanning the 17p13.1 region that incorporates the *TP53* locus was demonstrated in the BM-1 cell genomes, although the copy number of this region was normal (Fig. 2c). This copy-number-neutral LOH (CN-LOH) suggested that second hit was not the deletion, but that both alleles of the *TP53* gene in the ASM cells carried an E62X mutation, possibly due to mitotic recombination. Surprisingly, an LOH of the rs1042522 SNP was also found in the dysgerminoma cells without the E62X mutation (Fig. 2b).

The V1846F missense variant of *TET2* (NM_001127208.3:c.5536G > T) was found from our analysis of a buccal mucosal sample from the current study patient to be a germline variant (Additional file 4: Fig. S3). In silico analysis further predicted this to be a deleterious variation (PolyPhen-2 = 0.852, SIFT = 0.034) that did not appear in the databases (Additional file 1: Table S3). Notably, *TET2* is one of the tumor suppressor genes associated with ASM [14–16]. Both *TET2* and *KIT* are located on the chromosome 4 and we found by microarray analysis that the copy number of chromosome 4 was decreased in BM-1 (Fig. 2c). To then investigate whether the inactivation of the *TET2* gene is involved in development of ASM, we examined the phase of the *TET2* variant and the rs1008658 SNP. According to the determined allele frequencies, the *TET2* mutation was linked to the chromosome harboring the C allele of rs1008658 and was lost in the tumor cells. We thus concluded that the *TET2* variant was not associated with the development of ASM, since this rare mutation had been lost in the ASM cells. Further to this, the presence of this rare *TET2* variant in the healthy father of our patient was consistent with it having a benign nature (Additional file 4: Fig. S3).

In addition to chromosomes 4 and 17, copy-number aberrations or LOHs were detected in chromosomes 1, 2, 6, 9, 12, 13, 14, 16, 18, 20, 21 and X in the BM-1 cells from our patient (Fig. 2c). No other mutations with the potential to be cancer driver candidates were detected by whole-exome sequencing of the affected regions of these chromosomes (Additional file 1: Table S3).

## Discussion

We have here presented an adolescent case of ASM which is a rare neoplasm at such a young age. Our female patient developed the ASM after prior treatment for a dysgerminoma. Significantly, the pathogenic *KIT* gene variant, D816A, was identified in both the dysgerminoma and BM samples in this case. *KIT* mutations are frequently found in the tumor cells of SM patients and are an important part of the established diagnostic criteria for ASM, but the most prevalent variant of these is the D816V mutation, which has been observed in > 60% of ASM patients [7]. The D816A mutation has been occasionally identified also in SM with an associated hematologic neoplasm [17–19] but not in ASM. *KIT* D816 mutations are also commonly observed in germ cell tumors, and have been found in one-third of ovarian dysgerminomas [13]. Notably however, the D816A mutation has not been reported previously in these tumors. Thus, the presence of the *KIT* D816A variant in both the ASM and dysgerminoma cells in our present patient is unlikely to be a coincidence. There are considerable number of reports describing the association between mastocytosis and germ cell tumor [20–24]. The etiological linkage between ASM and a preceding germ cell tumor caused by a *KIT* D816 mutation is further supported by a similar prior report of an ASM patient carrying *KIT* D816V who had previously had an ovarian germ cell tumor harboring this same mutation [25]. Another similar case of an ovarian germ cell tumor carrying a *KIT* D816H mutation was also recently reported in which the chemotherapy was complicated by the development of SM with chronic myelomonocytic leukemia harboring this same mutation [26].

**Fig. 3:**
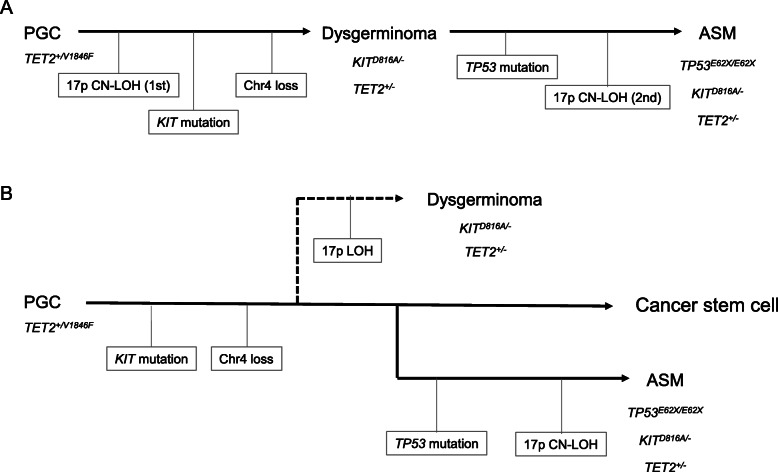
Schema for two possible mechanisms of ASM development in the study patient. **a** The first CN-LOH of 17p occurred as an incidental event in the PGCs. Subsequently, a cell which acquired the *KIT* mutation and lost one copy of chromosome 4 without that mutation was selected and gave rise to a dysgerminoma. After treatment for that lesion, a residual tumor cell differentiated into a mast cell and acquired the *TP53* mutation in one allele. Finally, a second CN-LOH event involving 17p led to the inactivation of the *TP53* gene in both alleles and caused the ASM. **b** A cancer stem cell carrying the *KIT* mutation arose from the PGCs. One of these cancer stem cells was fated to become the lineage for the dysgerminoma, which underwent a 17p LOH. Another cancer stem cell which acquired the *TP53* mutation was fated to cause the ASM. A cell of this tumor lineage underwent a 17p CN-LOH which inactivated both *TP53* alleles

One simple hypothesis to explain our current findings is clonal evolution (Fig. 3a). In brief, the *KIT* D816A variation initially induces the development of ovarian dysgerminoma. Although the tumor was removed by surgical resection, minimally residual cells that differentiated into hematologic cells possibly underwent subsequent biallelic mutation of the *TP53* gene, resulting in malignant transformation. Our microarray analysis indicated a complex karyotype and suggested the mast cells underwent repeated genetic rearrangements, although an abnormal karyotype is less common in ASM [5, 27, 28]. *TP53* is a less frequently affected gene in advanced SM [3], but is the most commonly affected gene in therapy-related myeloid neoplasms [29, 30]. The chemotherapy for the previous dysgerminoma in our present patient may have contributed to the mutation induction in a residual tumor cell and then expansion of these mutated mast cells, resulting in the younger onset of ASM in this case.

It is noteworthy that the LOH at the *TP53* locus was present both in the dysgerminoma and ASM, but that the ASM had the CN-LOH. The ASM cells were found to carry a biallelic homozygous E62X mutation of the *TP53* gene, suggesting that the CN-LOH was established by mitotic recombination after acquisition of the *TP53* mutation. However, the dysgerminoma was found to carry a 17p LOH without *TP53* mutation, suggesting that the 17p LOH in the dysgerminoma and ASM were independent events. A possible alternative hypothesis therefore is that the dysgerminoma and ASM originated from a common cancer stem cell harboring the *KIT* D816A mutation and developed independently (Fig. 3b). Briefly, the deletion of 17p possibly occurred in the dysgerminoma lineage. After treatment for this lesion, another cancer stem cell acquired the *TP53* mutation and underwent chromosome rearrangements, which evolved into the origin of another tumor lineage and caused the ASM.

Previously reported experiments using murine cells expressing pathogenic *KIT* mutants have demonstrated that the loss of one copy of *Tet2* accelerates mast cell growth [15, 31]. Although the effect of the *TET2* V1846F variation in tumorigenesis is not clear, cells with only one normal *TET2* allele may have a growth advantage under KIT D816A expression, even after deletion of the *TET2* V1846F allele in our current patient, suggesting that the *TET2* V1846F variant was unlikely to have affected the tumor progression.

Our current genetic analyses were useful in not only reaching a conclusive diagnosis but also for determining the treatment strategies in our study patient. Although the patient’s condition had not progressed during the current treatment and the genetic alternations in her bone marrow were not among the negative prognostic indicators reported previously [32], the comprehensive cancer-related genetic alterations that were detected in this case in addition to the *KIT* mutation suggested that a tyrosine kinase inhibitor would not be sufficient to achieve remission. Notably in this regard, the multikinase inhibitor midostaurin is not currently available in Japan. An allogenic bone marrow transplantation from an HLA 1-locus mismatched sibling donor is being planned for our study patient.

## Conclusions

Our current cytogenomic analyses demonstrated that mast cells causing the ASM shared a common origin with the dysgerminoma, suggesting that a careful follow-up should be required for the dysgerminoma patients after treatment.

## Supplementary Information

**Additional file 1: Table S1**. PCR primers used in this study. **Table S2.** BM-1-specific candidate variants of cancer-related genes analyzed by Mutect2. **Table S3.** Candidate variants of cancer-related genes identified in the PB of the study patient.

**Additional file 2: Figure S1.** Genetic analyses of the study patient. (A) PCR-direct sequencing of the *KIT* mutation site in the BM-2 sample. (B) Real-time quantitative PCR of the *KIT* gene. The *SNX25* gene is located on the 4q35.1. Ratio to the healthy control (mean ± SD) obtained from the two independent experiments are shown. (C) Cloning and sequence analysis of the PCR products from the BM-1.

**Additional file 3: Figure S2**. Integrative Genomics Viewer image of the next-generation sequencing reads of the *TP53* mutation site. The arrow indicates the transcriptional direction of the *TP53* gene. The ‘A’ at the E62X site and ‘C’ in the common SNP are the complementary bases of ‘T’ and ‘G’ in the context of gene coding, respectively.

**Additional file 4: Figure S3.** PCR-direct sequencing of the *TET2* mutation site in the PB, BM-1, dysgerminoma, and buccal mucosa samples from the patient, and PB samples from her parents and a healthy control.

## Data Availability

The datasets used and/or analyzed during the current study are available from the corresponding author on reasonable request.

## Abbreviations

AFP: α-fetoprotein
ASM: Aggressive systemic mastocytosis
BM: Bone marrow
CN-LOH: Copy-number-neutral loss of heterozygosity
LOH: Loss of heterozygosity
PB: Peripheral blood
PGC: Primordial germ cells
SM: Systemic mastocytosis
SNP: Single nucleotide polymorphism
VAF: Variant allele frequency
