# Supplementary material for: An aggressive systemic mastocytosis preceded by ovarian dysgerminoma
Source: BMC Cancer. 2020 Nov 27;20:1162. doi: 10.1186/s12885-020-07653-z (PMC7693501; doi:10.1186/s12885-020-07653-z)
Supplement: Supplementary file 2 — Additional file 2: Figure S1. Genetic analyses of the study patient. (A) PCR-direct sequencing of the KIT mutation site in the BM-2 sample. (B) Real-time quantitative PCR of the KIT gene. The SNX25 gene is located on the 4q35.1. Ratio to the healthy control (mean ± SD) obtained from the two independent experiments are shown. (C) Cloning and sequence analysis of the PCR products from the BM-1. [file 12885_2020_7653_MOESM2_ESM.pptx]

## Slide 1
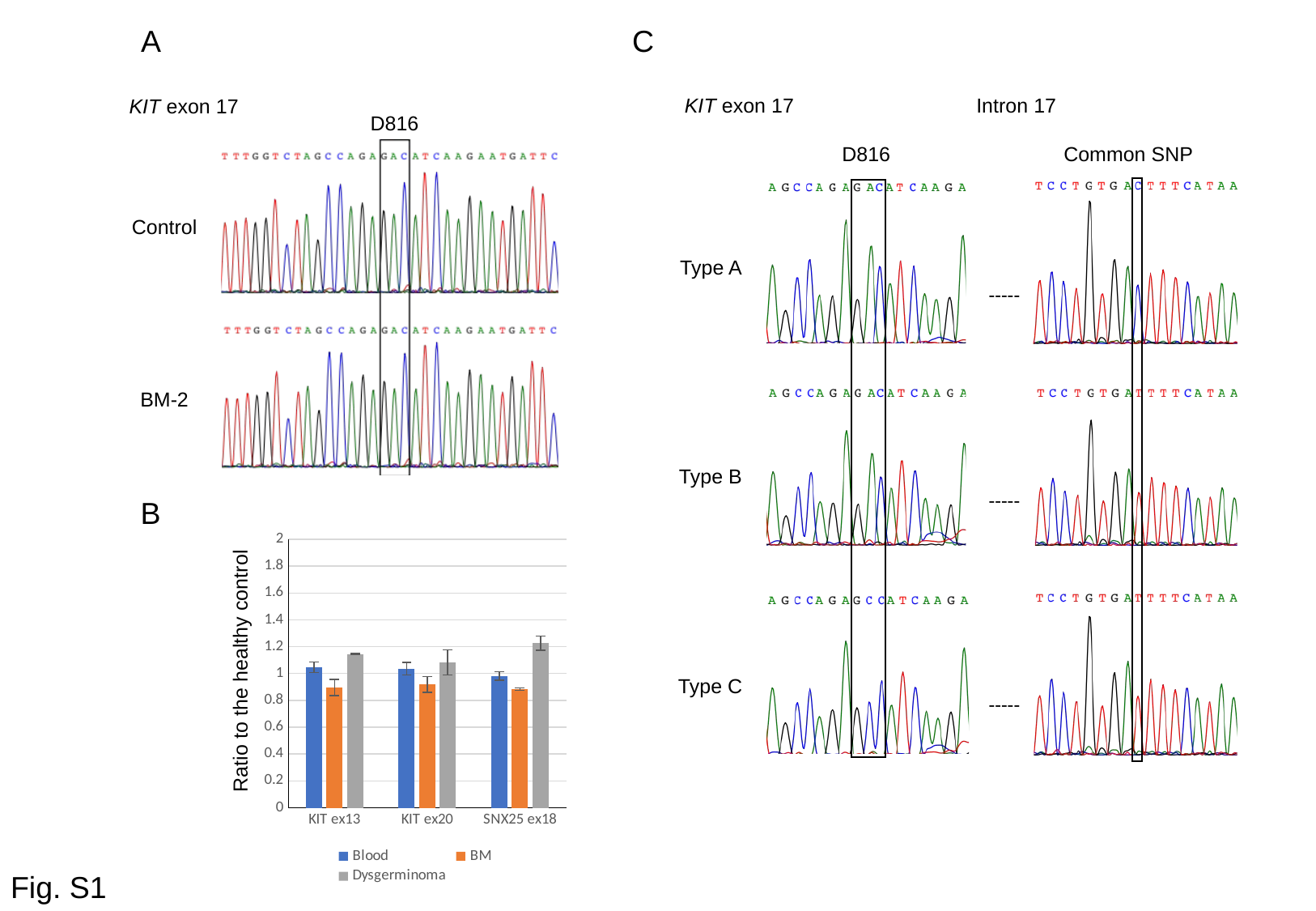

A
C
KIT exon 17
Intron 17
KIT exon 17
D816
D816
Common SNP
Control
Type A
-----
BM-2
Type B
-----
B
### Chart
| Category | Blood | BM | Dysgerminoma |
|---|---|---|---|
| KIT ex13 | 1.046471122768326 | 0.8952388568247436 | 1.1447901578923754 |
| KIT ex20 | 1.036506431689571 | 0.9183271515947065 | 1.0817812108741431 |
| SNX25 ex18 | 0.9824566476008494 | 0.8826234251833653 | 1.2255379585088488 |Ratio to the healthy control
Type C
-----
Fig. S1
