# Supplementary figures and images for: An aggressive systemic mastocytosis preceded by ovarian dysgerminoma
Source: BMC Cancer. 2020 Nov 27;20:1162. doi: 10.1186/s12885-020-07653-z (PMC7693501; doi:10.1186/s12885-020-07653-z)

## Slide 1
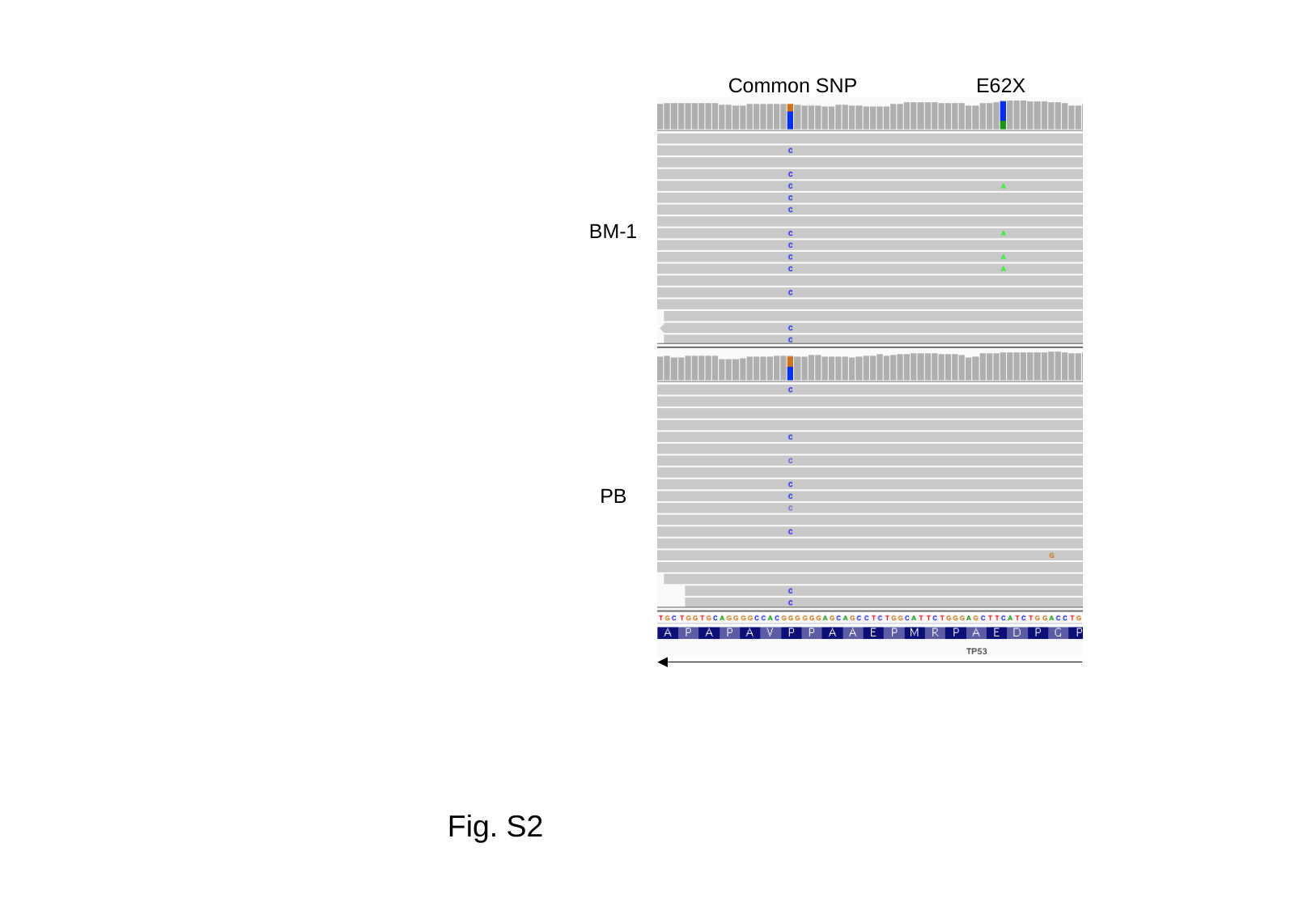

Common SNP
E62X
BM-1
PB
Fig. S2

Supplement: Supplementary file 3 — Additional file 3: Figure S2. Integrative Genomics Viewer image of the next-generation sequencing reads of the TP53 mutation site. The arrow indicates the transcriptional direction of the TP53 gene. The ‘A’ at the E62X site and ‘C’ in the common SNP are the complementary bases of ‘T’ and ‘G’ in the context of gene coding, respectively. [file 12885_2020_7653_MOESM3_ESM.pptx]
