# Supplementary material for: An aggressive systemic mastocytosis preceded by ovarian dysgerminoma
Source: BMC Cancer. 2020 Nov 27;20:1162. doi: 10.1186/s12885-020-07653-z (PMC7693501; doi:10.1186/s12885-020-07653-z)
Supplement: Supplementary file 4 — Additional file 4: Figure S3. PCR-direct sequencing of the TET2 mutation site in the PB, BM-1, dysgerminoma, and buccal mucosa samples from the patient, and PB samples from her parents and a healthy control. [file 12885_2020_7653_MOESM4_ESM.pdf]

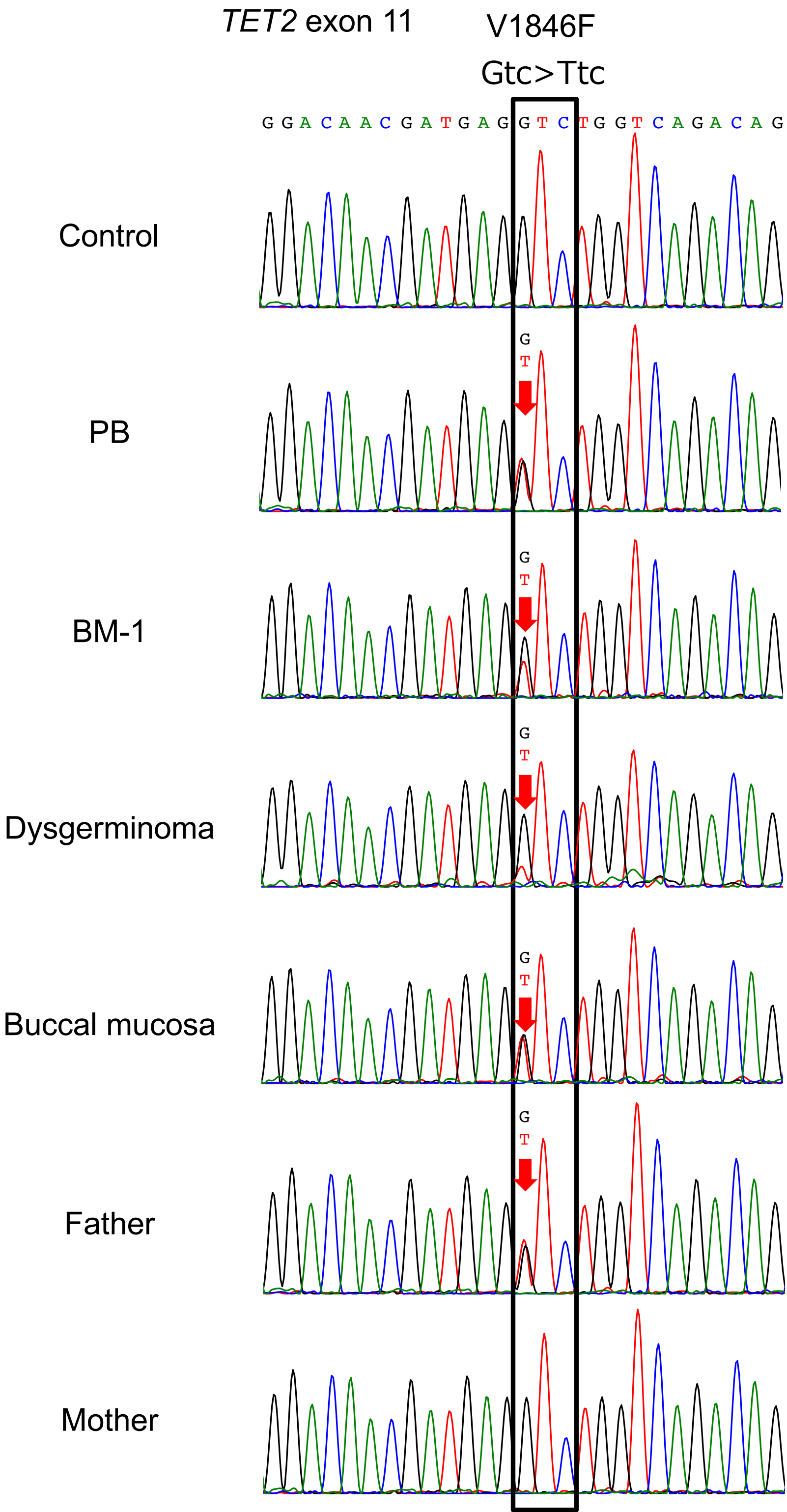

TET2 exon 11
V1846F
Gtc>Ttc
G G A C A A C G A T G A G G T C T G G T C A G A C A G
Control
PB
BM-1
Dysgerminoma
Buccal mucosa
Father
Mother

Fig. S3
